# Translational Relevance of Advanced Age and Atherosclerosis in Preclinical Trials of Biotherapies for Peripheral Artery Disease

**DOI:** 10.3390/genes15010135

**Published:** 2024-01-22

**Authors:** Keith A. Webster

**Affiliations:** 1 Vascular Biology Institute, University of Miami, Miami, FL 33146, USA; kwebster@med.miami.edu; 2 Department of Ophthalmology, Baylor College of Medicine, Houston, TX 77030, USA

**Keywords:** gene therapy, cell therapy, peripheral artery disease, critical limb ischemia, preclinical models, clinical trials

## Abstract

Approximately 6% of adults worldwide suffer from peripheral artery disease (PAD), primarily caused by atherosclerosis of lower limb arteries. Despite optimal medical care and revascularization, many PAD patients remain symptomatic and progress to critical limb ischemia (CLI) and risk major amputation. Delivery of pro-angiogenic factors as proteins or DNA, stem, or progenitor cells confers vascular regeneration and functional recovery in animal models of CLI, but the effects are not well replicated in patients and no pro-angiogenic biopharmacological procedures are approved in the US, EU, or China. The reasons are unclear, but animal models that do not represent clinical PAD/CLI are implicated. Consequently, it is unclear whether the obstacles to clinical success lie in the toxic biochemical milieu of human CLI, or in procedures that were optimized on inappropriate models. The question is significant because the former case requires abandonment of current strategies, while the latter encourages continued optimization. These issues are discussed in the context of relevant preclinical and clinical data, and it is concluded that preclinical mouse models that include age and atherosclerosis as the only comorbidities that are consistently present and active in clinical trial patients are necessary to predict clinical success. Of the reviewed materials, no biopharmacological procedure that failed in clinical trials had been tested in animal models that included advanced age and atherosclerosis relevant to PAD/CLI.

## 1. Introduction

Peripheral artery disease (PAD), the third leading cause of atherosclerotic morbidity after coronary heart disease and stroke, refers primarily to lower limb ischemia and includes asymptomatic lower limb PAD, intermittent claudication (IC), and critical limb ischemia (CLI). PAD affects approximately 6% of adults globally and about 8.5 million people in the United States [1,2,3]. Incidence is markedly age-dependent, increasing from about 5% in subjects aged 40 to 44 years to >12% at age 70 to 74, with some studies reporting IC in up to 35% of patients over 50 years of age, and 1% to 2% with CLI [4,5,6]. Medical therapies to ameliorate hyperlipidemia, hypertension, and hyperglycemia combined with thrombolytics or fibrinolytics reduce morbidity and mortality related to cardiovascular events but have limited impact on PAD symptoms or disease progression [7,8]. Other major risk factors for PAD include smoking, sedentary lifestyle, high BMI, and elevated systemic inflammatory markers [4,9]. Currently, only supervised walking programs and the phosphodiesterase type 3 (PDE3) inhibitor cilostazol, an antiplatelet vasodilator, provide symptomatic benefits including improved pain-free walking time (PFWT). Surgical or endovascular revascularization remain primary interventions for lifestyle-limiting claudication and CLI, but this option is not available for up to 40% of CLI patients [10,11]. Despite two decades of intense basic and translational research efforts to develop gene and/or cell therapies, no new treatments have been approved in the US or EU, leading some to question whether PAD/CLI is even amenable to such biotherapies, especially gene therapy. Positive results of preclinical studies along with partial successes in some clinical trials of gene therapy, and broader successes of cell therapies, provide important information on the most promising pro-angiogenic biopharmacological strategies, but reveal larger protocol and translation flaws, and limited predictive value of preclinical models. This review discusses the failures of earlier gene therapy clinical trials, the possible reasons for such failures, impacts on ongoing biopharmacology research for PAD, and avenues to resolve the dilemmas of translation and optimization. The broad interpretation that gene therapy for PAD/CLI has failed should be tempered by the approvals and ongoing application of plasmid (p)VEGF and pHGF, respectively, in Russia and Japan, and the significant, albeit limited, efficacies seen for gene therapy on some clinical parameters such as rest pain and ulcer healing. Such spasmodic efficacy suggests inadequate strategies of implementation rather than fundamental misconceptions related to the approach and invites further analysis and optimization.

## 2. Literature Review

### 2.1. Meta-Analyses of Early Clinical Trials of Genes and Cells

**Figure 1:**
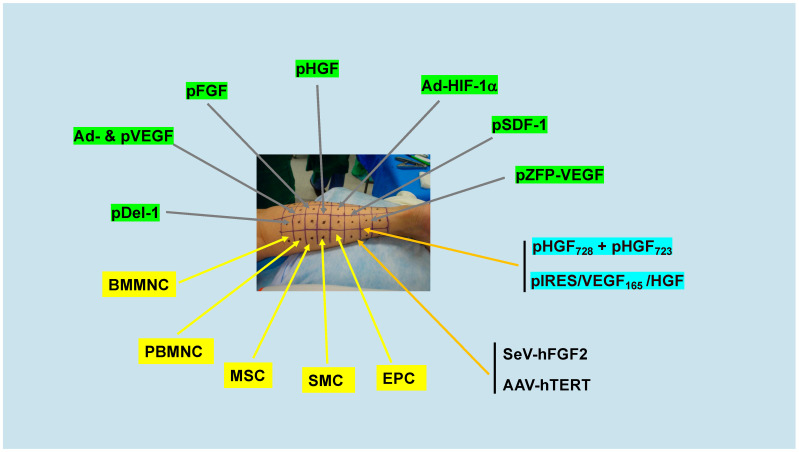
Gene and cell targets of therapeutic angiogenesis. Green boxes show target pro-angiogenic genes that have been tested in Phase I-III RCTs delivered IA or IM as proteins, cDNA plasmids or cDNA adenovirus. Yellow boxes indicate autologous cells also delivered IA or IM. Aqua boxes indicate ongoing trials of combination genes including two isoforms of human HGF (pHGF_728_ and pHGF_723_) and a bicistronic plasmid vector expressing human VEGF165 and HGF (pIRES/VEGF165/HGF). Unboxed denotes ongoing RCTs with viral delivery of human FGF2 by IM of Sendai virus and IM injections of human telomerase reverse transcriptase (hTERT) by AAV.

Whereas early meta-analyses of gene and stem cell clinical trials of patients with PAD and CLI confirm universal safety of the procedures, efficacy to enhance blood flow, prevent or delay amputation and/or death, and improve PFWT and/or quality of life have been underwhelming. Genes tested in these trials include VEGF, FGF, HIF-1α, HGF, Del-1, SDF-1, and ZFP-VEGF (an engineered zinc finger transcription factor of VEGF-A), delivered by plasmid or adenoviral vectors (reviewed in: [7]). Cell therapies include bone marrow mononuclear cells (BMMNC), peripheral blood mononuclear cells (PBMNC), mesenchymal stem cells (MSC), endothelial progenitor cells (EPC), and smooth muscle cells (SMC). Figure 1 shows the genes and cells that have undergone positive preclinical evaluation and progressed to clinical trials by IA or IM delivery of recombinant human proteins, plasmids, or viral vectors. In the first meta-analysis of Phase 2 randomized, controlled clinical trials (RCT) of gene and cell therapies for PAD/CLI, De Haro et al. [12] reported that therapeutic angiogenesis significantly improved clinical outcomes including peak PFWT relative to placebo in both PAD and CLI cohorts. The authors concluded that gene and cell therapies were safe, well tolerated, and conferred significant efficacy for both PAD and CLI patients (see Table 1 and Refs. [13,14,15,16,17,18] therein). Four years later, using similar analyses that included updated trial results, Steiner and Hammer [19] concluded from 12 PAD/CLI gene therapy RCTs and a total of 1494 patients that endpoints were not significantly improved in the majority of studies. This meta-analysis showed neither significant benefit nor harm for gene therapy across all-cause mortality, amputations, or ulcer healing, and no differences in outcomes between patients with PAD or CLI. The meta-analysis included the Talisman 201 Phase II and TAMARIS Phase III RTCs of plasmid (p)FGF1 as well as pHGF, Ad- and pVEGF, pDel-1 and Ad-HIF1α, (see Table 2 and Refs. [20,21,22,23,24,25,26,27] therein); TAMARIS, with 525 CLI patients, was the largest worldwide gene therapy trial ever conducted [20,28]. Subsequent meta-analyses confirmed these results, including a study with 1988 PAD/CLI patients that reported no improvement of amputation-free survival, major amputation, or death by gene therapy relative to placebo [29]. These and other disappointing results that culminated with the withdrawal in 2016 of the multinational Phase III AGILITY RTC of pHGF for CLI (NCT02144610), brought this phase of translational research to an end and led to predictions that clinical trials of therapeutic angiogenesis by gene therapy for PAD were over [7]. 

By conferring more comprehensive stimuli involving multiple cytokines and growth factors with autocrine and paracrine angiogenic effects, cell therapy has been considered potentially superior, although more expensive and technically more demanding, than proteins or genes for promoting angiogenesis and tissue salvage in PAD. TACT (therapeutic angiogenesis using cell transplantation) was the first major cell clinical trial for PAD [18] that compared BMMNC with PBMNC. The study reported safety and significantly greater improvements in ABI, rest pain, and PFWT at 24 weeks in the BMMNC group. PROVASA (intra-arterial administration of BMMNC in patients with CLI), a Phase II RTC of intra-arterial BMMNC versus placebo, reported no difference in ABI or limb salvage but significant improvements in ulcer healing and rest pain [26]. The largest JUVENTAS (rejuvenation EPC via transcutaneous intra-arterial supplementation) trial of CLI patients with intra-arterial EPCs versus placebo, reported no significant differences in major amputation rate, quality of life, rest pain, ABI, or TcPO2 [27]. In a 2017 meta-analysis of autologous cell therapy that included 19 RCTs (837 patients), 7 nonrandomized trials (338 patients), and 41 noncontrolled studies (1177 patients), Rigato et al. [30] reported that cell therapy reduced the risk of amputation by 37%, and improved amputation-free survival and wound healing by 18% and 59%, respectively. Cell therapy also significantly increased ABI and TcPO2, and reduced rest pain. The authors noted that the efficacy of end points was no longer significant in placebo controlled RCTs and disappeared in RTCs with a low risk of bias. In a 2018 meta-analysis of RCTs of autologous stem cell therapy in CLI, Xie et al. [31] reported significantly improved ABI, TcO2, PFWT, as well as ulcer healing, reduced amputation rates, and increased angiogenic scores in the cell therapy group relative to controls. The analysis concluded that while cell therapy may be safe and effective, higher quality and larger RCTs are still required to support clinical application. In another recent (2019) meta-analysis of autologous stem cell therapy [32] that included 27 RCTs and 1186 patients, Gao et al. reported significantly improved healing of ulcers, ABI, TcO2, and PFWT, reduction of rest pain, and minor amputation rates, but no significant improvement in major limb salvage. The authors acknowledged high risk of bias and low-quality of evidence outcomes and concluded that autologous stem cell therapy may have a positive effect on “no-option” patients with PAD but did not significantly impact major limb amputation. In a meta-analysis of autologous cell therapy for CLI including 12 RCTs and 630 patients, Pu et al. [33] reported significantly improved total and major amputation rates, ABI, TcO2, and rest pain score compared with placebo or standard care but no change in all-cause death or ulcer size and concluded that autologous cell therapy conferred benefit to CLI patients in terms of limb salvage, perfusion, and rest pain alleviation. The results of the meta-analyses of autologous cells are summarized in Table 3. 

In their review of angiogenic cell therapy for CLI up to and including 2021 data, Beltrán-Camacho et al. [34], concluded that 20 years of clinical trials with autologous stem cells confirms safety and promising efficacy but due to high variability between studies and low to moderate quality of data, it remains unclear which cells, doses, or routes of administration are optimal. One possible problem cited by several authors is that most studies are supported by sponsors with potentially conflicted interests and reporting is often incomplete and/or subjective. However, despite this, most authors agree on the safety and feasibility of cell therapies and concur that they represent a promising approach for no option CLI, a population that represents 50% of CLI patients [35,36]. Ongoing trials are also testing secreted products such as exosomes that avoid the potential hazards and regulatory/technical demands of live autologous cell therapy and optimizing cells by preconditioning and/or genetic engineering [37,38,39]. Although, autologous cell-based approaches now appear to show greater promise than gene therapy, the procedures are more expensive, labor intensive, and technically demanding.

### 2.2. Recent and Ongoing Clinical Trials of Genes and Cells II

With the exception of ongoing trials of pVM202 and JVS-100 (see below), the 2017 prediction by Iyler and Annex [7] that “termination of the multinational Phase III AGILITY trial may well end gene therapy trials of therapeutic angiogenesis for peripheral arterial disease”, appears to have been substantially validated within the US. Despite insufficient evidence of efficacy for approval in the US, pVEGF-165 (Neovasculgen) was approved to treat CLI patients by the Russian Ministry of Healthcare in 2011 and the Ukraine equivalent in 2013. The decisions were based on positive safety/efficacy findings of pre-clinical studies and a Phase IIB/III clinical trial [40,41,42]. Similarly, pHGF (Collategene) was approved by the Japanese Ministry of Health to treat ulcers in no-option CLI patients in 2019. This approval was based on positive results from preclinical and clinical studies including a Phase III RCT [24,43,44,45,46]. Clinical studies of pHGF containing two isoforms of human pHGF (HGF_728_ and HGF_723_) to treat PAD are also ongoing in the US and China. HI-PAD, a Phase II RTC of pVM202 in the US was initiated in 2018 (NCT03363165) and a Phase III trial of NL003 (HGF-X7) in China was initiated in 2019 (NCT04274049). Awaited outcome results include wound healing, rest pain, and limb salvage. In a related Phase III RCT of patients with painful diabetic peripheral neuropathy, Kessler et al. [47] recently reported significant alleviation of pain by pVM202 in one arm of the trial. A Phase II RCT in Poland assessed safety and efficacy of a bicistronic plasmid vector expressing human VEGF_165_ and HGF (pIRES/VEGF165/HGF) by intramuscular injections in diabetic CLI patients [48]. Preliminary results indicate safety and efficacy with significantly increased ABI, reduced rest pain, and improved vascularization assessed by computed tomography angiography. In Beijing, China, a dose escalation, safety, and tolerability Phase I clinical trial of human FGF-2 delivered via intramuscular injection of Sendai virus (SeV-hFGF2) to CLI patients, initiated in 2018, is underway (NCT03668353). In another Phase II RCT, Shishehbor et al. reported that gene therapy with JVS-100 (pSDF-1) conferred no significant improvement in outcomes of patients undergoing revascularization [49]. The Libella gene therapy trial in Colombia is an ongoing Phase I safety and tolerability trial of intravenous adeno-associated virus (AAV) expressing the human telomerase reverse transcriptase (AAV-hTERT) in CLI patients (NCT04110964). Telomerases protect against age- and cell division-dependent telomere shortening, thereby delaying senescence and preserving gene function. hTERT was shown to augment VEGF-A activity and enhance the regenerative properties of endothelial progenitor cells (EPC) in vitro and in vivo. Adenovirus (Ad)-hTERT combined with Ad-VEGF was shown to enhance angiogenesis, vascular remodeling, and perhaps muscle regeneration in a rat CLI model [50], and the hope is that AAV-hTERT ameliorates telomere dysfunction and augments vascular regeneration in elderly PAD patients. A parallel trial will test AAV-hTERT in Alzheimer’s patients. Recruitment for the Libella trials commenced in 2019.

### 2.3. Predictive Value of Preclinical Models 

Despite the Russian and Japanese approvals, respectively, of pVEGF and pHGF, and the ongoing trials of pVM202 and JVS-100, the universal failure of large Phase III US-based clinical trials of gene therapy for CLI has markedly dampened enthusiasm for the technology and prompted speculation on the reasons for the failures [34,51,52]. Possible explanations include: (1) use of single angiogenic growth factors that generate immature leaky capillaries reminiscent of pathological angiogenesis [53]; (2) limitation of most interventions to stimulate only angiogenesis when revascularization may require enhanced arteriogenesis and vasculogenesis; (3) insufficient level, duration, targeting, and regulation of transgene expression; and (4) inadequate predictive value of preclinical models that do not include comorbidities of PAD/CLI patients [1,2,3,54,55]. Plasmids have been the vectors of choice for all major trials so far, whereas AAV and lentiviral vectors may provide higher levels of sustained, regulable expression [56]. However, the inadequacy of preclinical models to accurately predict clinical translation stands out as a major reason for the failed gene therapy trials. Two properties of the most used mouse models that fail to replicate clinical targets include: (1) acute ischemia by femoral artery ligation and excision (FAL) that does not replicate occlusion by progressive atherosclerotic narrowing by arterial plaque, and (2) absence of comorbidities that determine the responsiveness of ischemic limb tissues to pro-angiogenic stimuli. Comorbidities of PAD/CLI patients that are rarely incorporated into mouse models include age, atherosclerosis, hypertension, hyperglycemia, hyperlipidemia, diabetes, smoking, sedentary lifestyle, and elevated systemic inflammation [51,57,58,59]. Of these, advanced age and severe atherosclerosis are the only comorbidities that are consistently present in patients with clinical PAD/CLI. Other comorbidities that exacerbate clinical PAD and are variously present in clinical trial subjects are usually well-controlled by standard pharmacology that includes antithrombotic drugs, and medications to treat dyslipidemia, hypertension, and diabetes. Such management reduces the risk of major adverse events (AMI and stroke) but does not alter the course of PAD progression or the outcome of biopharmacological (gene/cell) clinical trials [1,3]. The average age of patients in the major Phase III clinical trials of gene and cell therapies for CLI is >70 years and all patients were symptomatic with leg pain and ulcers secondary to chronic ischemia caused by atherosclerotic plaque build-up in the in-flow blood vessels [60]. For example, the mean age of patients in the TAMARIS trial was 70 years (equivalent to 25-month C57BL/6 mice), wherein atherogenic stenoses were present in >95% of infrainguinal arteries, 66% of thigh arteries, and 94% of arteries below the knee. In total, 70% of patients had more than one diseased artery, 80% were hypertensive, 60% hypercholesterolemia, 61% former or current smokers, 53% diabetic, and 18% obese. Similar hemodynamic and angiographic patterns were typically reported in all regional areas, as well as in diabetic and non-diabetic patients. Therefore, the vasculatures of aged CLI patients with chronic, severe atherosclerosis are unlikely to replicate the responses to angiogenic stimulation of young healthy mice with acute ischemic damage.

### 2.4. Age and Atherosclerosis in Mouse CLI Models 

Multiple hindlimb FAL models have been described wherein the intrinsic aptitude for vascular regeneration and functional recovery is dependent on the mouse strain [57,61,62,63,64]. Whereas most models are made acutely ischemic via double FAL with FA excision, new models more accurately replicate occlusion in clinical PAD [58,65,66]. Strain- and age-dependent responses to angiogenic stimulation, including roles of circulating PBMNC and BMMNC, have been described [67,68]. In the latter study, Bosch-Marce et al. compared intrinsic perfusion recovery rates of young (2 months), mid-aged (8 months), and old-aged (20 months) C57BL/6 mice after FAL. Old-age mice salvaged only 40% of limbs and achieved < 30% perfusion compared with young mice. The authors attributed the differences to loss of mobilization of VEGFR2+/CD34+ angiogenic cells and deceased levels of pro-angiogenic cytokines after FAL in the ischemic limbs of aged mice. Reductions of cytokines included ANGPT1 & 2 (<10%), HIF-1α (<25%), MCP-1(<10%), PLGF (<10%), SCF (<10%), and SDF-1 (~10%). These trends were confirmed in other studies that reported similar ischemia, but <50% recovery of limb function in 18-month versus 3-month-old mice after FAL, and diminished arteriogenesis in the ischemic hind limbs of older mice [64,69]. Collaterals in aged mice are less able to remodel and enlarge in response to FAL because of deficient eNOS production and increased susceptibility of ECs and SMCs to apoptosis [70]. HIF-1α levels are decreased in the ischemic hind limbs of aged mice and correlate with reduced angiogenic factors, lower recruitment of angiogenic cells, and loss of perfusion recovery after FAL [70]. Deficiency of Klotho, an anti-aging gene in mice confers a phenotype equivalent to human aging that includes short lifespan, stunted growth, vascular calcification, and atherosclerosis [71,72,73]. Mice with heterozygous deficiency of the klotho gene show impaired neovascularization and perfusion recovery after FAL with markedly decreased nitric oxide release and reduction of BMMNCs [74].

Atherosclerosis progression involves the accumulation of lipids, inflammatory cells, and smooth muscle cells in arterial walls that culminate in necrosis, fibrosis, and calcification [75]. Systemic inflammation is associated with severe PAD and atherothrombotic narrowing during PAD promotes changes in the circulatory system and tissues including NO-dependent compensatory responses, angiogenesis and arteriogenesis, and detrimental effects including microvascular dysfunction, myopathy, fibrosis, and tissue necrosis [76,77]. PAD is associated with reduced calf skeletal muscle area and density, increased calf muscle fat infiltration, increased oxidative stress, impaired mitochondrial activity, and smaller myofibers [78,79]. ApoE-knockout (ApoE-/-) mice mimic traits of human atherosclerosis including inflammation and metabolism and are commonly used to simulate the effects of atherosclerosis and dyslipidemia [80,81]. Studies from multiple groups, including the author’s, have demonstrated that the evolution of atherosclerosis in ApoE-/- mice is paralleled by infiltration of inflammatory cells and progressive loss of mobility of BMMNCs with reduced levels of angiogenic factors including SDF-1 [82,83,84,85]. ApoE-/- C57BL/6 mice develop significant atherosclerotic lesions in the ascending aorta, carotid, femoral, and popliteal arteries [86,87,88], coincident with delayed recovery from ischemia and stunted response to angiogenic therapy [89,90]. Couffinhal et al. [91] first showed that recovery of young C57BL/6 ApoE-/- mice from hindlimb FAL was markedly attenuated relative to WT controls. Capillary density, CD-31, and VEGF that were significantly reduced in the ApoE-/- group paralleled increased infiltration of inflammatory cells, leukocytes, macrophages, and T-lymphocytes. Similar results were reported by Xie et al., effects that were partially reversed by delivery of ZFP-32E, a zinc finger DNA-binding transcription factor of VEGF [92]. Using a FAL model that included Western diet and 7-month aged ApoE-/- mice, Lejay et al. [93] reported more severe mitochondrial dysfunction and increased oxidative stress after FAL of ApoE-/- mice relative to control WT mice. Blunted responses of ApoE-/- mice to angiogenic stimuli have been variously attributed to dysregulated expression of miRs that regulate angiogenesis and vasculogenesis in PAD [94,95,96,97,98,99]. Peck et al. [61] subjected 8-month-old ApoE-/- fed a normal diet to exercise training after FAL and documented responses that mimic CLI patients. They proposed that such aged ApoE-/- mice represent a more appropriate hindlimb ischemia model to accurately evaluate therapeutic strategies for human PAD/CLI. By regulating inflammation and EC angiogenesis, respectively, miR-146b and miRNA-27b are implicated in the suppressed angiogenic responses of ApoE-/- mice [100,101]. Together, results from multiple sources concur that age and atherosclerosis create toxic environments for vascular regeneration, and their absence represent a shortcoming of therapeutic angiogenesis preclinical trials to date. By supporting optimization on a relevant background, inclusion of age and atherosclerosis is expected to provide more accurate predictions of clinical success.

### 2.5. Recent and Ongoing Preclinical Trials of Genes, MiRs, and NO-Donors

**AGGF1** (angiogenic factor with G-patch and Forkhead-associated domain-1) binds the integrin α5β1 receptor on ECs [102] and is the earliest known regulator of multipotent hemangioblast specification, regulating hematopoiesis and differentiation of endothelial lineages [103]. AGGF1 regulates EC proliferation, adhesion, migration, and capillary tube formation [104,105] and promotes angiogenesis as potently as VEGF-A [106]. In hindlimb ischemia mouse models, pAGGF1 promoted therapeutic angiogenesis more efficiently than FGF2 [107,108]. Transplantation of AGGF1-transduced EPCs conferred limb salvage, reperfusion, and exercise tolerance in high-fat diet and db/db diabetic mouse hindlimb ischemia models [109]. AGGF1 also binds and regulates VSMC phenotypic switching, proliferation, and migration [110], and drives therapeutic angiogenesis through a pathway of integrin α5β1, FAK, Src, and AKT signaling. The authors predict that AGGF1, through its roles in regulating vasculogenesis, angiogenesis, and vascular development, represents a promising target for clinical development to provide a more effective therapy for PAD/CLI [111]. 

**Anti-angiogenic VEGF165b** is an alternative spliced isoform of VEGF-A that is increased in ischemic muscle [2] and competes with pro-angiogenic isoforms of VEGF-A for binding to VEGFR2 [112]. VEGF165b is a weak agonist of VEGFR2, and the interaction lacks the downstream signaling required for an angiogenic response, making it a competitive inhibitor of the VEGFR2-AKT-ERK-eNOS-NO angiogenic pathway [113,114,115]. By suppressing NO production, elevated VEGF165b in muscles of PAD/CLI patients may contribute to the absence of clinical benefit seen in VEGF-A clinical trials or of NO supplementation by L-arginine in PAD patients. With the rationale that PAD/CLI patients have chronically reduced responses to NO signaling and thence therapeutic angiogenesis, Kuppuswamy et al. [2] recently showed that delivery of an anti-VEGF165b antibody significantly enhanced perfusion and increased microvascular density in three mouse PAD models with suppressed NO production including T2D and eNOS knock-out mice. The authors conclude that VEGF165b is a potential therapeutic target for patients with PAD where the VEGFR2-eNOS-NO pathway is impaired. The same group recently identified the IL-21 receptor (IL-21R) as causally linked to the differential responses of C57BL/6 versus Balb/c mice to hindlimb ischemia. High IL-21R expression in ECs of ischemic hindlimbs in C57BL/6 mice coincided with enhanced perfusion recovery whereas low IL-21R expression in Balb/C correlated with sustained perfusion deficit and greater tissue loss during HLI [61]. Genetic support linking the IL-21R with human PAD prompted the authors to target IL-21R for nitric oxide-independent angiogenesis in PAD [1]. 

**MiR-15 and -16** belong to an extended miR-16 family that bind to Tie2 mRNA coding sequences (CDSs) and regulate angiogenesis by targeting VEGFR2 and FGFR1 [116]. The miRs are conserved between humans and mice [117]. MiR-15a and -16 are increased in serum and circulating proangiogenic cells (PACs) of CLI patients wherein serum concentrations predict amputation at 1-year post-revascularization [118]. Ex vivo transfection with miR-15a/16 inhibitors increase the potential of human PACs to induce therapeutic angiogenesis in mouse PAD models [118] and therapeutic angiogenesis is impaired in mice with *miR-15a* gene knock-in [119]. Local adenoviral delivery of a 15a/16 decoy increased Tie2 levels in ischemic skeletal muscle, improved perfusion recovery, and reduced toe necrosis. The results support further development of Ad-Decoy-15a/16 to treat human PAD/CLI.

**MiR-150**. Using next-generation sequencing and quantitative reverse transcription polymerase chain reaction analyses. Desjarlais et al. [120] reported that decreased levels of the proangiogenic microRNA miR-150 in ApoE-/- mice conferred decreased Src, eNOS, and Akt activities that was mechanistically associated with inefficient neovascularization following FAL. The effects were normalized by forced expression of an miR-150 mimic that the authors propose to represent a novel therapeutic strategy to improve ischemia-induced neovascularization in atherosclerotic conditions. However, this model does not represent the average age or severity of atherosclerosis of human CLI.

**AAV-PFKFB3** delivery was shown to salvage limbs, increase perfusion, and improve muscle contractile function following FAL in BALB/c mice [121]. The model mimics CLI patients wherein compromised mitochondria and inflexible metabolism exacerbate myopathy. The authors report that the glycolytic enzyme 6-phosphofructo-2-kinase/fructose-2,6-bisphosphatase 3 (PFKFB3) was markedly induced in transgenic mice with defective mitochondrial metabolism caused by accumulated mutations in mtDNA, and this conferred resistance to ischemic myopathy by enhancing glycolysis and maintaining ATP in ischemic muscle. Muscles from CLI patients were shown to contain lower PFKFB3 relative to normal or claudication muscles and decreased glycolytic flux capacity. The results support reduced glycolytic flux as a common characteristic of failing CLI patient limb skeletal muscle that may be responsive to gene therapy with AAV-PFKFB3. 

**MPC-1011,** an NO-donor, stimulates angiogenesis and arteriogenesis and improves hindlimb ischemia via a cGMP-dependent pathway involving VEGF and SDF-1α [122]. Atherosclerosis-impaired NO production and associated vascular dysfunction is well-documented in PAD patients and animal models [123,124,125,126,127,128]. Preclinical studies and small clinical trials confirm increased PFWT in PAD patients via NO augmentation with beetroot juice, derivates of dark chocolate, MitoQ, a mitochondria-targeted antioxidant, and PDE-V inhibitor sildenafil [129,130,131,132,133].

**ACAT 1/2 inhibitors.** High-intensity statins are recommended for patients with peripheral artery disease (PAD) and meta-analyses have shown that CLI patients benefit from statin therapy with significantly lower amputation rates (~25%) and fewer fatal events compared with control groups without statin therapy [134,135]. Attempts to augment this effect include inhibition of acyl-coenzyme A:cholesterol acyltransferase (ACAT; EC 2.3.1.26) enzymes that regulate cholesterol homeostasis by esterifying the 3-hydroxyl position of cellular free cholesterol with a fatty acid-CoA, creating cholesteryl ester (CE). ACAT1 is responsible for CE accumulation in macrophage foam cells and its inhibition was predicted to reduce cholesterol accumulation in atherosclerotic lesions. Positive anti-atherosclerosis activity of ACAT1 inhibitors in mouse and rabbit models supported three multi-center placebo controlled RCTs of two different ACAT1 inhibitors. However, human trials reported no significant improvement in atheroma volume regression, and in some cases, significantly increased major cardiovascular events in treated vs. placebo [136,137]. The reasons for the absence of clinical success are unclear, but there were indications of adverse effects of ACAT inhibition in some animal studies involving mitochondrial dysfunction, cytotoxicity, proinflammatory effects, and apoptosis, that the authors suggest may be alleviated by switching to ACAT2 inhibitors [137].

**NLRP3 inflammasome** stimulation by cellular stress activates caspase-1 and cleavage of pro-inflammatory cytokines IL-1β and IL-18, that trigger an inflammatory response. Quantitative trait locus mapping and molecular technologies in mice have identified new genetic loci (*Ath28*, *Ath22*, *Ath26*) and associated genes (*Soat1*, *Gpnmb*, *AKR Pycard*) associated with NLRP3 inflammasomes that determine macrophage phenotypes and associated atherosclerosis plaque and pathology [138,139,140]. Targeted inhibition of the NLRP3 inflammasome pathway represents another ongoing promising approach to slow atherosclerosis.

## 3. Summary and Conclusions

While the positive albeit variable results of cell therapies for PAD/CLI encourage further testing to optimize and consolidate cell types and procedures, the limited efficacies revealed by large clinical trials of gene therapy have led to reduced enthusiasm for continued development of this approach. However, gene therapy clinical trials were based on preclinical models that lacked predictive value for clinical translation, and much evidence suggests that more predictive models would identify different protocols with increased likelihood of clinical success. At minimum, such models would preclude expensive, time, and labor-intensive clinical development of products that were predestined to fail. Importantly, all Phase III gene therapy trials were limited to plasmid gene delivery, whereas more efficient viral delivery (AAV/Lentivirus) allows higher expression, targeting, and defined duration of transgene expression, as demonstrated in a PAD model and previously reviewed by the author’s group [56,141,142] and in other indications [143,144,145]. Newer gene therapy approaches, including those listed above, may benefit from preliminary testing in preclinical models that include advanced age and atherosclerosis (ApoE-/- mice) prior to clinical development. Such an application, while arduous, is not as labor intensive, time consuming, and expensive as the build-up and implementation of clinical trials; by some estimates the cost of TAMARIS was >$25M. While FDA IND approvals for cardiovascular indications require two animal species, relevant co-morbidities are not required and not routinely included. 

Advanced age and atherosclerosis create toxic environments that render host tissues, vascular beds, and resident cells resistant to vascular regeneration and tissue salvage. Heightened local and systemic inflammation, suppressed expression of angiogenic and arteriogenic growth factors and cytokines, impaired NO production, dysregulated miRs that drive angiogenesis and arteriogenesis, microvascular dysfunction, increased oxidative stress, and impaired metabolic regulation including mitochondrial and glycolytic dysfunctions may contribute to muted responses to vasculogenesis. The effects worsen in parallel with age and severity of atherosclerosis. CLI patients in the major failed clinical trials of gene therapy were of advanced age (mean of 70 years) with severe atherosclerosis. To our knowledge, none of the angiogenic genes subject to clinical trial were tested in preclinical models that included equivalent backgrounds of advanced age and atherosclerosis. Mouse PAD/CLI models that include age and/or atherosclerosis show markedly reduced responses to both gene and cell treatments. Application of more inclusive models, including surgical techniques that more closely mimic the target population of PAD patients, is predicted to provide much needed information to optimize both gene and cell therapies for clinical application. 

## Figures and Tables

**Table 1:** RCTs from 2000–2007 used for meta-analysis. For a complete list of studies see De Haro et al. [12] and Iyer and Annex [7]. IA: intra-arterial; IM: intramuscular; N/C: no significant change; QoL: quality of life. rFGF2: recombinant fibroblast growth factor 2. All treatments are deemed safe with indication of efficacy in rFGF-2 and BMMNC trials.

Author	Phase	Treatment	Treatment/CTRL	Major Findings
Lazarous et al. [13]	I	IA rFGF-2	13/6	Safe. Increased calf blood flow at 6 months in treatments.
Rajagopalan et al. [14] (RAVE)	II	IM Ad2- VEGF121	15/3	Safe. N/C ABI, PFWT, QoL.
Lederman et al. [15] (TRAFFIC)	II	IA rFGF-2	116/58	Safe. Improved PFWT at 90 days; early improved ABI.
Makinen et al. [16]	II	IA Ad2- and pVEGF165	35/19	Ad antibodies. Improved vascularity both treatments; N/C ABI or Rutherford class vs control.
Grossman et al. [17] (DELTA)	II	IM pDel-1 + poloxamer 188	52/53	N/C PFWT ABI, claudication compared with control poloxamer 188 alone.
Tateishi-Yuyama et al. [18] (TACT)	II	IM BMMNC or PBMNC	25 unilateral 22 bilateral	Safe. Improved ABI, TcO2, PWT, increased collateral vessels in BM-MNC vs. PBMNC.

**Table 2:** RCTs from 2008–2015 used for meta-analysis. For a complete list of studies see Hammer A, Steiner S [19] and Iyer and Annex [7]. IA: intra-arterial; IM: intramuscular; N/C: no significant change; QoL: quality of life. rFGF2: recombinant fibroblast growth factor 2. All treatments are deemed safe with indications of efficacy especially in pHGF and BMMNC trials.

Author	Phase	Treatment	Treatment/CNTR	Major Findings
Belch et al. [20] (TAMARIS)	III	IM pFGF1	259/256	N/C amputation or death
Van Huyan et al. [21]	II	IA + IM BMMNC	12/15	Improved PFWT and ABI
Powell et al. [22] (HGF STAT)	II	IM pHGF	56/23	Increased TcPO2; N/C TBI, ABI, wound healing
Nikol et al. [23] (TALISMAN)	II	IM pFGF1	59/66	Improved rest pain, QoL, amputation; N/C wound healing
Shigematsu et al. [24]	III	IM pHGF	27/13	Improved rest pain, ulcer size, QoL; N/C ABI or amputation
Creager et al. [25]	II	IM AdHIF1α	213/76	N/C PFWT, QoL, ABI
Walter et al. [26] (PROVASA)	II	IA BMMNC	19/21	Improved ulcer healing, rest pain, N/C ABI, amputation, death
Teraa et al. [27] (JUVENTAS) (2015)	II	IA EPC	81/79	N/C amputation, death, ABI, ulcer size, QoL, rest pain, TcPO2

**Table 3:** Meta-analyses of autologous cell therapy conclude that the procedures are safe with evidence of efficacy on multiple outcomes including reduced amputation rates and QoL parameters. Variability of methods, reporting, and quality of data do not yet allow a determination on suitability for translation to clinical practice.

Author	Title	Major Findings
Rigato et al. [30] (2017)	Autologous Cell Therapy for Peripheral Arterial Disease: Systematic Review and Meta-Analysis of Randomized, Nonrandomized, and Noncontrolled Studies.	Autologous cell therapy may reduce the risk of major amputation, improve the probability of wound healing, and amputation-free survival, ameliorate pain and functional capacity. Results of the primary analysis were confirmed and strengthened by secondary analysis. No change in all-cause mortality.
Xie et al. [31] (2018)	Autologous Stem Cell Therapy in Critical Limb Ischemia: A Meta-Analysis of Randomized Controlled Trials.	Cell therapy significantly increased the probability of ulcer healing, angiogenesis, and reduced amputation rates. ABI and PFWT were significantly improved. Higher quality and larger RCTs are required to support clinical application.
Gao et al. [32] (2019)	Autologous stem cell therapy for peripheral arterial disease: a systematic review and meta-analysis of randomized controlled trials.	Improved healing rate of ulcers, ABI, TcO2, and PFWT; reduction in amputation rate and rest pain scores, no significant improvement in major limb salvage. High risk of bias and low-quality evidence of outcomes. Larger, placebo controlled, RCT are needed.
Pu et al. [33] (2022)	A meta-analysis of randomized controlled trials on therapeutic efficacy and safety of autologous cell therapy for atherosclerosis obliterans.	No-option CLI patients show significantly improved total amputation, major amputation, ABI, TcO2, and rest pain score compared with standard care. No effect on all-cause death or ulcer size.
Beltrán-Camacho et al. [34] (2021)	Current Status of Angiogenic Cell Therapy and Related Strategies Applied in Critical Limb Ischemia	Cell therapy may represent an alternative for no-option CLI. Variability between trials is high, reflecting a lack of consensus on cell dose, cell types or sources, administration routes, parameters to define outcome efficacy, or cohorts themselves. Further investigation is required to better understand mechanism. Much work is needed to translate to clinical practice.

## Abbreviations

Peripheral artery disease: PAD; critical limb ischemia: CLI; pain-free walking time: PFWT; plasmid: p; vascular endothelial growth factor: VEGF; fibroblast growth factor: FGF; hepatocyte growth factor: HGF; hypoxia-inducible factor-1-alpha: HIF-1α; developmental endothelial locus-1: Del-1, stromal-derived factor 1: SDF-1; bone marrow mononuclear cells: BMMNC; peripheral blood mononuclear cells: PBMNC; mesenchymal stem cells: MSC; endothelial progenitor cells: EPC; smooth muscle cells: SMC; intra-arterial: IA; intramuscular: IM; quality of life: QoL; randomized, controlled clinical trials: RCT; ankle brachial ratio: ABI, total carbon dioxide:TcO2; adeno-associated virus: AAV; adenovirus: Ad; femoral artery ligation: FAL; angiopoietins: ANGPT; monocyte chemoattractant protein-1: MCP-1; placental growth factor: PLGF; stem cell factor: SCF; nitric oxide: NO.
